# Estrogen differentially regulates transcriptional landscapes of preoptic and arcuate kisspeptin neuron populations

**DOI:** 10.3389/fendo.2022.960769

**Published:** 2022-08-25

**Authors:** Balázs Göcz, Szabolcs Takács, Katalin Skrapits, Éva Rumpler, Norbert Solymosi, Szilárd Póliska, William H. Colledge, Erik Hrabovszky, Miklós Sárvári

**Affiliations:** ^1^ Laboratory of Reproductive Neurobiology, Institute of Experimental Medicine, Budapest, Hungary; ^2^ János Szentágothai Doctoral School of Neurosciences, Semmelweis University, Budapest, Hungary; ^3^ Centre for Bioinformatics, University of Veterinary Medicine, Budapest, Hungary; ^4^ Department of Biochemistry and Molecular Biology, Faculty of Medicine, University of Debrecen, Debrecen, Hungary; ^5^ Department of Physiology, Development and Neuroscience, University of Cambridge, Cambridge, United Kingdom

**Keywords:** fertility, kisspeptin neuron, RNA-seq, neuropeptides, dense-core vesicle, transcription factors, reproduction

## Abstract

Kisspeptin neurons residing in the rostral periventricular area of the third ventricle (KP^RP3V^) and the arcuate nucleus (KP^ARC^) mediate positive and negative estrogen feedback, respectively. Here, we aim to compare transcriptional responses of KP^RP3V^ and KP^ARC^ neurons to estrogen. Transgenic mice were ovariectomized and supplemented with either 17β-estradiol (E2) or vehicle. Fluorescently tagged KP^RP3V^ neurons collected by laser-capture microdissection were subjected to RNA-seq. Bioinformatics identified 222 E2-dependent genes. Four genes encoding neuropeptide precursors (*Nmb, Kiss1, Nts, Penk*) were robustly, and *Cartpt* was subsignificantly upregulated, suggesting putative contribution of multiple neuropeptides to estrogen feedback mechanisms. Using overrepresentation analysis, the most affected KEGG pathways were neuroactive ligand-receptor interaction and dopaminergic synapse. Next, we re-analyzed our previously obtained KP^ARC^ neuron RNA-seq data from the same animals using identical bioinformatic criteria. The identified 1583 E2-induced changes included suppression of many neuropeptide precursors, granins, protein processing enzymes, and other genes related to the secretory pathway. In addition to distinct regulatory responses, KP^RP3V^ and KP^ARC^ neurons exhibited sixty-two common changes in genes encoding three hormone receptors (*Ghsr, Pgr, Npr2*), GAD-65 (*Gad2*), calmodulin and its regulator (*Calm1, Pcp4*), among others. Thirty-four oppositely regulated genes (*Kiss1, Vgf, Chrna7, Tmem35a*) were also identified. The strikingly different transcriptional responses in the two neuron populations prompted us to explore the transcriptional mechanism further. We identified ten E2-dependent transcription factors in KP^RP3V^ and seventy in KP^ARC^ neurons. While none of the ten transcription factors interacted with estrogen receptor-α, eight of the seventy did. We propose that an intricate, multi-layered transcriptional mechanism exists in KP^ARC^ neurons and a less complex one in KP^RP3V^ neurons. These results shed new light on the complexity of estrogen-dependent regulatory mechanisms acting in the two functionally distinct kisspeptin neuron populations and implicate additional neuropeptides and mechanisms in estrogen feedback.

## Introduction

The kisspeptin neuropeptide family includes hormones of varying amino acid length released from the prohormone product of the *Kiss1* gene. Kisspeptin producing neurons mediate the effect of estrogens to GnRH neurons *via* the KiSS-1 receptor and play indispensable role in the regulation of GnRH/LH pulsatility and estrogen feedback mechanisms. Inactivating mutations of *KISS1R*, which encodes the KiSS-1 receptor (1, 2) or *KISS1* itself (3), cause hypogonadotropic hypogonadism in humans. These reproductive defects can be replicated in knockout mouse models (2, 4, 5).

Most kisspeptin producing neurons reside in two areas of the rodents’ hypothalamus. One population, KP^ARC^ neurons, are localized in the arcuate nucleus (ARC). Their majority co-express neurokinin B and dynorphin, and are, therefore, called KNDy neurons. KP^RP3V^ neurons are mainly located in the anteroventral periventricular nucleus (AVPV) and the periventricular nucleus (PeN) of the preoptic area, and co-express galanin (6, 7), met-enkephalin (6) and some markers for dopamine (8, 9), GABA (10) and glutamate (10) phenotypes. The two kisspeptin neuron populations innervate different cellular domains of GnRH neurons (11). KP^ARC^ neurons innervate distal dendrons at the median eminence while KP^RP3V^ neurons contact the soma and proximal dendrites in the preoptic area. Kisspeptin exerts stimulatory effects on GnRH neurons and triggers GnRH secretion into the portal circulation at the median eminence, which in turn, increases the synthesis and secretion of gonadotropins in the anterior pituitary (12). In females, KP^ARC^ and KP^RP3V^ neurons mediate the negative and positive estrogen feedback, respectively, on gonadotropin secretion. Earlier transcriptomic studies provided partial insight into the molecular phenotype of KP^ARC^ and KP^RP3V^ neurons. In these Drop-seq studies, cells of the ARC (13) and the preoptic area (14) have been categorized based on their transcriptional profile. KP^ARC^ neurons have been described as KISS1/TAC2 while KP^RP3V^ neurons as dopaminergic cells, suggesting that the two populations display distinct molecular phenotypes.

Hypothalamic kisspeptin neurons express nuclear hormone receptors including estrogen receptor α (ERα), which enable them to respond to changes in circulating estrogen levels. Estrogens are robust transcriptional regulators of *Kiss1* (15). In KP^ARC^ neurons, E2 inhibits *Kiss1* expression through a non-classical estrogen receptor mechanism, whereas in KP^RP3V^ neurons, E2 activates *Kiss1* transcription *via* the classic mode of action (16). In a recent study, we dissected the genome-wide transcriptional responses of KP^ARC^ neurons to E2 (17) and identified thousands of E2-dependent genes. Here, we used the same animals as in the case of KP^ARC^ neurons with surgical ovariectomy model with or without E2 replacement. From each, we collected three hundred pooled, fluorescently labelled KP^RP3V^ neurons by laser-capture microdissection (LCM). Transcriptomes of KP^RP3V^ neurons were determined by Illumina-based RNA-seq in the same way as in our recent KP^ARC^ neuron study (17) and then, bioinformatic analysis was performed using stringent criteria to generate the list of E2-regulated genes without low expressing and statistically non-significant genes. The detailed E2-dependent transcriptome of KP^ARC^ neurons has been published recently from our laboratory. Sequencing files, placed in a public repository (BioProject with the accession number of PRJNA686688), were re-analyzed with the same criteria to compare the KP^RP3V^ and KP^ARC^ neuron transcriptomes. The comparative analysis focused on neuropeptides, granins and genes of the secretory pathway, because of the presence of a large number of changes in these categories. Finally, the markedly different E2-driven responses of the two cell types were attributed to different transcriptional mechanisms revealed in KP^RP3V^ and KP^ARC^ neurons.

## Materials and methods

### Animals

Animal experiments were carried out in accordance with the Institutional Ethical Codex, Hungarian Act of Animal Care and Experimentation (1998, XXVIII, section 243/1998) and the European Union guidelines (directive 2010/63/EU). All efforts were made to minimize potential pain or suffering, and to reduce the number of animals used. Procedures were approved by the Institutional Animal Care and Use Committee. Young adult (day 60-80) female mice (n=6) were housed under standard conditions (lights on between 06:00 and 18:00 h, temperature 22 ± 1°C, chow and water *ad libitum*) in the animal facility of the Institute of Experimental Medicine. The E2-dependent transcripts of KP^RP3V^ neurons were identified in KP-Cre/ZsGreen mice generated by crossing Kiss1-Cre (18) males with females of the Ai6(RCL-ZsGreen) indicator strain (The Jackson Laboratory, JAX No. 007906) as described previously (17). The paper which reported generation of the Kiss1-Cre transgenic mouse provided evidence that 80-90% of fluorescently labelled cells expressed kisspeptin in the ARC (18).

### Surgical ovariectomy and subsequent E2 replacement

We have recently published a detailed protocol for the dissection of KP^ARC^ neurons (17). The same protocol was used here to dissect KP^RP3V^ neurons from the preoptic area. In brief, all mice were first anesthetized and ovariectomized (OVX) bilaterally. On post-ovariectomy day 9, the animals were implanted subcutaneously with a single silastic capsule (Sanitech, Havant, UK; l=10 mm; id=1.57 mm; od=3.18 mm) containing either 100 μg/ml E2 (Sigma Chemical Co., St Louis, MO) in sunflower oil (OVX+E2 group, n=3) or oil vehicle (OVX+Veh group, n=3) (19). Four days later, mice were sacrificed between 09:00-11:00 am. We have recently established that this E2 regimen resulted in 7.59 pg/mL serum E2 levels (high diestrus/proestrus range) and 7.58-fold uterine hypertrophy (17).

### LCM-assisted dissection of KP^RP3V^ neurons

We followed our recently published protocol for LCM-assisted dissection of fluorescent neurons. In brief, treated KP-Cre/ZsGreen mice (n=6) were perfused transcardially with 0.5% formaldehyde, followed by 20% sucrose. Brains were snap-frozen and tissue blocks containing the preoptic area were dissected. Then, coronal sections were cut from the preoptic area, collected onto PEN slides (Membrane Slide 1.0 PEN, Carl Zeiss, Göttingen, Germany) and air-dried in the cryostat chamber. Formaldehyde-fixed sections, containing fluorescent KP^RP3V^ neurons were treated sequentially with 50% EtOH, n-butanol:EtOH and xylene substitution:n-butanol. Three hundred KP-Cre/ZsGreen neurons were microdissected from 12-µm-thick preoptic sections of each mouse. Microdissected cells were pressure-catapulted into 0.5 ml tube caps (Adhesive Cap 200, Carl Zeiss), pooled and were stored at -80 °C until RNA extraction.

### RNA sequencing

Total RNA samples from KP^RP3V^ neurons were prepared with the Arcturus Paradise Plus RNA Extraction and Isolation Kit (Applied Biosystems, Waltham, MA, USA), and converted into RNA-seq libraries with the TrueSeq Stranded Total RNA Library Preparation Gold kit (Illumina, San Diego, CA, USA). Although the TrueSeq Stranded kit was optimized for 100 ng input RNA, a recent study found that it generates reliable libraries from as little as ng amounts of RNA (20). Total RNA extracted from 300 KP^ARC^ neurons provided sufficient amount of cDNA input for sequencing (17). For DNA fragment enrichment, our protocol used 16, instead of 15 cycles recommended by the manufacturer. Sequencing was performed on Illumina NextSeq500 instrument using the NextSeq500/550 High Output v2.5 kit (75 cycles). Sequencing files were deposited to BioProject with accession number of PRJNA847063.

### Bioinformatics

Following FastQC quality control, sequencing reads with low quality bases were removed using Trimmomatic 0.39 (settings: LEADING:3, TRAILING:3, SLIDINGWINDOW:4:30, MINLEN:50). Sequencing reads were mapped to the mm100 mouse reference genome using STAR (v 2.7.3a) (21), which resulted in an average overall alignment rate of 74.9 ± 3.5%. Read summarization and gene level quantification were performed by featureCounts (subread v 2.0.0) (22). Raw read counts were normalized and processed further with the packages of edgeR (23) and DESeq2 (24). EdgeR and DESeq2 calculated count per million (cpm) values and identified differentially expressed genes, respectively. Changes in mRNA expression were quantified by log2 fold change (log_2_FC). P values were corrected by the method of Benjamini (25) to take multiple testing into account. In differential expression analysis with DESeq2 we applied the basemean>20, p.adj<0.05 cutoffs to generate the list of E2-regulated genes without low-expressing and statistically non-significant genes. Genes were assigned to KEGG (26) signaling pathways by the R package KEGGREST (Dan Tenenbaum (2019): KEGGREST: Client-side REST access to KEGG., R package version 1.26.1). Overrepresentation analysis (ORA) (27) was performed by the clusterProfiler (28) R packages. All program packages for differential expression analysis and pathway analysis were run in the R environment (R2020). E2-dependent transcription factors were identified using functional classification with the Animal Transcription Factor DataBase (29). Putative transcription factors were double-checked using UniProt (https://www.uniprot.org) website. Listed transcription factors fulfilled the criteria to have ‘transcription factor activity’ GO molecular function, and experimental evidence at protein level.

## Results

### RNA-seq of KP^RP3V^ neurons reveals 222 E2-dependent genes

**Figure 1:**
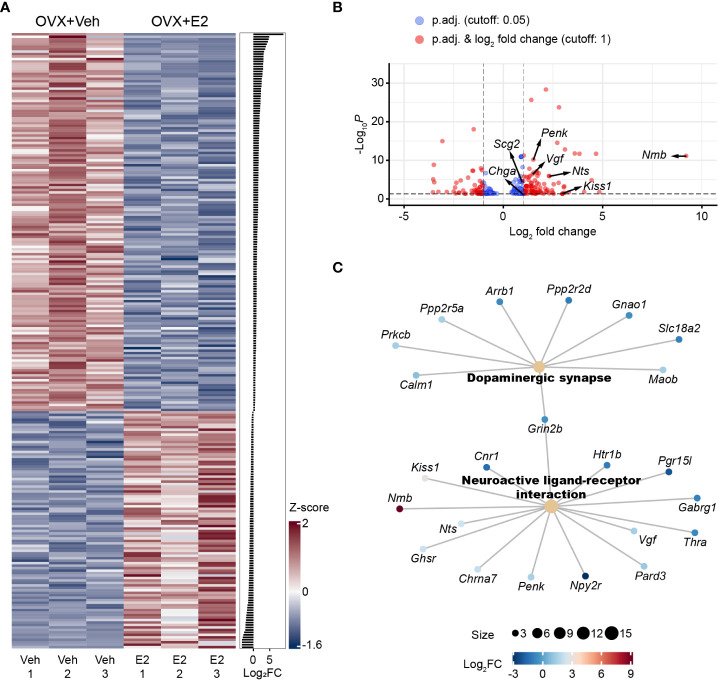
Estrogenic regulation of the KP^RP3V^ neuron transcriptome. Heat map of all E2-dependent transcripts. Transcripts were arranged by size of fold change (FC). We used z-score values to illustrate the size of transcriptional changes, and the values are color coded. z-score is calculated from the CPM value, the mean CPM and the standard deviation of CPM values in a given experimental group **(A)**. Volcano plot reveals 132 regulatory changes that exceed |log_2_FC| 1.0. Transcriptional changes of neuropeptides (*Nmb, Kiss1, Nts, Penk*) and granins (*Chga, Scg2, Vgf*) were marked **(B)**. Overrepresentation analysis (ORA) of E2-dependent genes identified significant changes in the dopaminergic synapse and the neuroactive ligand-receptor interactions KEGG pathways. The number of genes in a given pathway is reflected in the size of the dot for the pathway. E2-induced changes of individual genes are color coded based on log_2_FC values **(C)**.

To examine estrogenic regulation of KP^RP3V^ neurons, we dissected and pooled fluorescent KP^RP3V^ neurons by LCM from OVX mice substituted with either oil or E2. Illumina-based RNA-seq was performed to determine the transcriptional landscape of KP^RP3V^ neurons at high physiological (7.59 pg/mL) and gonadectomy E2 levels which latter is below 0.3 pg/mL (30). Initial DESeq2 analysis identified 203 E2-regulated genes in KP^RP3V^ neurons with the p.adj<0.05 cutoff. P.adj values are highly sensitive to the number of comparisons which can severely compromise the detection power for true positives (31, 32). Noisy, low-expression genes were shown to have adverse impact on the power of statistics in RNA-seq studies (32). Given that these genes likely have relatively minor effect on kisspeptin neuron biology, we improved the power of DeSeq2 analysis by filtering out low-expression genes. Using the basemean>20 and p.adj<0.05 cutoffs, we identified 10,623 transcripts, 247 of which were E2-dependent (**Supplementary Table/ Table 1**) including 222 protein coding genes. The 222 genes contained 45 new changes that were not included in the list unfiltered to low basemean. The heat map of E2-regulated transcripts showed disparate expression in the two experimental groups (**Figure 1A**). Among the 222 protein coding genes, 142 were up- and 80 were downregulated. The most robust upregulation was seen in the case of *Nmb* encoding neuromedin B (**Figure 1B**). Highly upregulated genes (log_2_FC>1) comprised additional neuropeptides (*Kiss1, Nts, Penk*) and a granin (*Vgf*), among others. *Cartpt* encoding neuropeptide CART was also highly upregulated, but the change did not reach statistical significance. ORA identified enrichment in changes of the dopaminergic synapse and neuroactive ligand-receptor interaction KEGG pathways (**Figure 1C**). Using gene ontology (GO) terms, ORA revealed significant enrichment of changes in the regulation of membrane potential (GO:0042391), synapse organization (GO:0050808), peptide transport (GO:0015833), hormone secretion (GO:0046879) GO categories, among others (**Supplementary Table/ Table 2**).

### Comparative analysis unveils disparate E2-driven transcriptional responses

**Figure 2:**
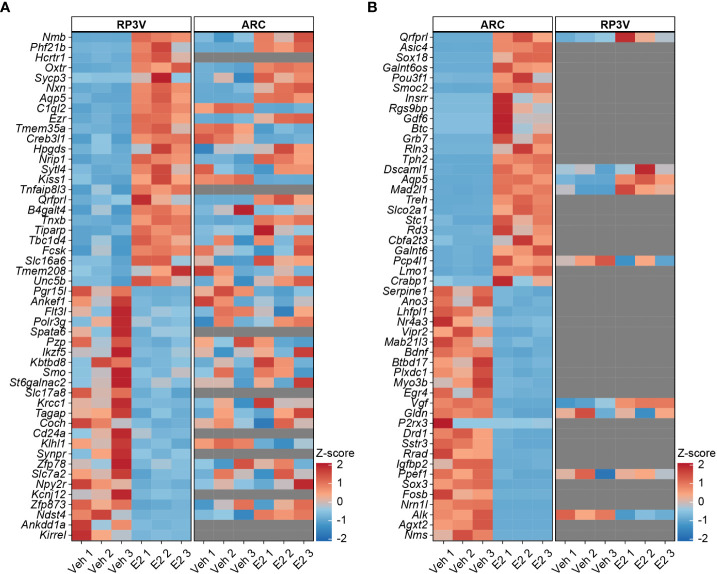
E2 differentially regulates the transcriptomes of preoptic and arcuate KP neurons. Heat map of the top 25 activated and top 25 inhibited genes in KP^RP3V^ neurons and behavior of the same genes in KP^ARC^ neurons **(A)**. Heat map of the top 25 activated and top 25 inhibited genes in KP^ARC^ neurons and their behavior in KP^RP3V^ neurons **(B)**.

For consistency, we re-analyzed our recently deposited RNA-seq data of KP^ARC^ neurons (17) with the same filtering which resulted in 1583 medium-to-high abundance E2-regulated genes. While 470 low-expressed genes were excluded by this filtering, the enhanced statistical power resulted in the identification of 48 newly identified genes in KP^ARC^ neurons. Compared to KP^RP3V^ neurons, the KP^ARC^ neurons showed much higher number and more robust transcriptional responses to E2. To display differences in estrogenic regulation of the two populations, we generated heat maps with the top 25 activated and top 25 suppressed genes in KP^RP3V^ neurons and illustrated in parallel expression of the same genes from KP^ARC^ neurons (**Figure 2A**). The top 25 activated and top 25 suppressed genes of KP^ARC^ neurons and their behavior in KP^RP3V^ neurons are shown similarly in **Figure 2B**. Markedly different responses of the two kisspeptin neuron populations to E2 prompted us to check the expression of major estrogen receptors. We detected abundant mRNA expression of *Esr1* encoding ERα in both KP^RP3V^ and KP^ARC^ neurons. However, we did not detect transcription of *Esr2* and *Gper1* encoding ERβ and G-protein coupled estrogen receptor, respectively.

### Despite disparate regulation there are ninety-six overlapping E2 target genes

**Figure 3:**
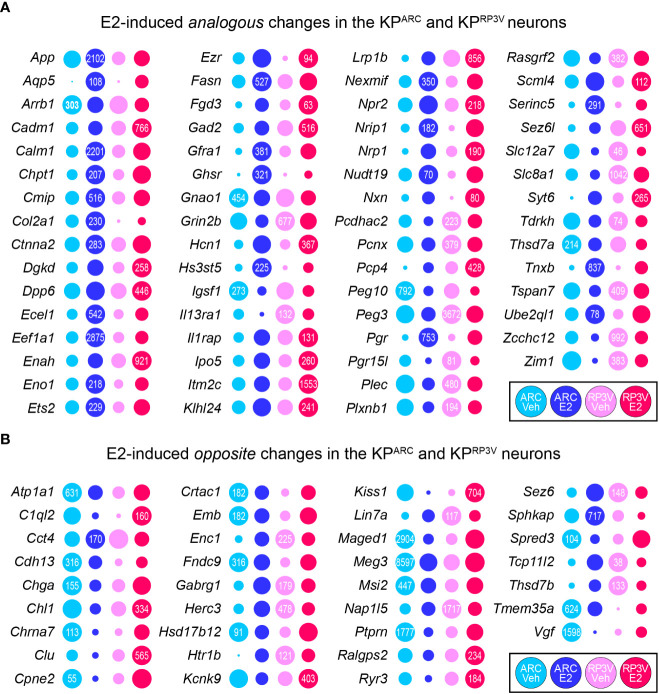
Overlapping E2 target genes in KP^RP3V^ and KP^ARC^ neurons. E2 regulated 62 genes in the same (**A**, analogous changes), and 34 genes in opposite direction (**B**, opposite changes). Numbers in the dots reflect transcript abundance in CPM units.

Although the E2-driven transcriptional responses were different, we found ninety-six overlapping genes with sixty-two analogous and thirty-four opposite changes in preoptic and arcuate kisspeptin neurons. The sixty-two genes, which were regulated in the same direction consisted of transcription factors, synapse associated genes and calcium signaling molecules, among others (**Figure 3A**). There were 25 genes that displayed |log_2_FC| >1.0 changes in both populations representing the highly responsive, common E2-dependent genes in kisspeptin neurons. Among highly expressed genes, E2 upregulated *App, Itm2c* (inhibits APP processing), *Calm1, Eef1a1*. E2 also increased expression of some synapse-associated genes including *Cadm1, Enah, Gad2, Syt6*, and decreased *Grin2b*. In addition, E2 enhanced mRNA expression of major calcium signaling molecules (*Pcp4, Calm1*) and pacemaker channel *Hcn1* in both cell populations. Thirty-four genes including *Kiss1* were oppositely regulated (**Figure 3B**), and several of them, regulated in a similar fashion as *Kiss1* (*Atp1a1, Chga*, *Vgf, Ptprn, Ralgps2*), were associated with neuropeptide secretion. Other oppositely regulated genes encoded proteins related to translational control (*Msi2*), calcium signaling (*Cpne2, Ryr3*), protein quality control (*Clu*), synaptic plasticity (*Cct4, Chl1*), cholinergic transmission (*Chrna7, Tmem35a*), among other functions.

### E2 activates neuropeptide precursor and granin genes in KP^RP3V^ neurons

**Figure 4:**
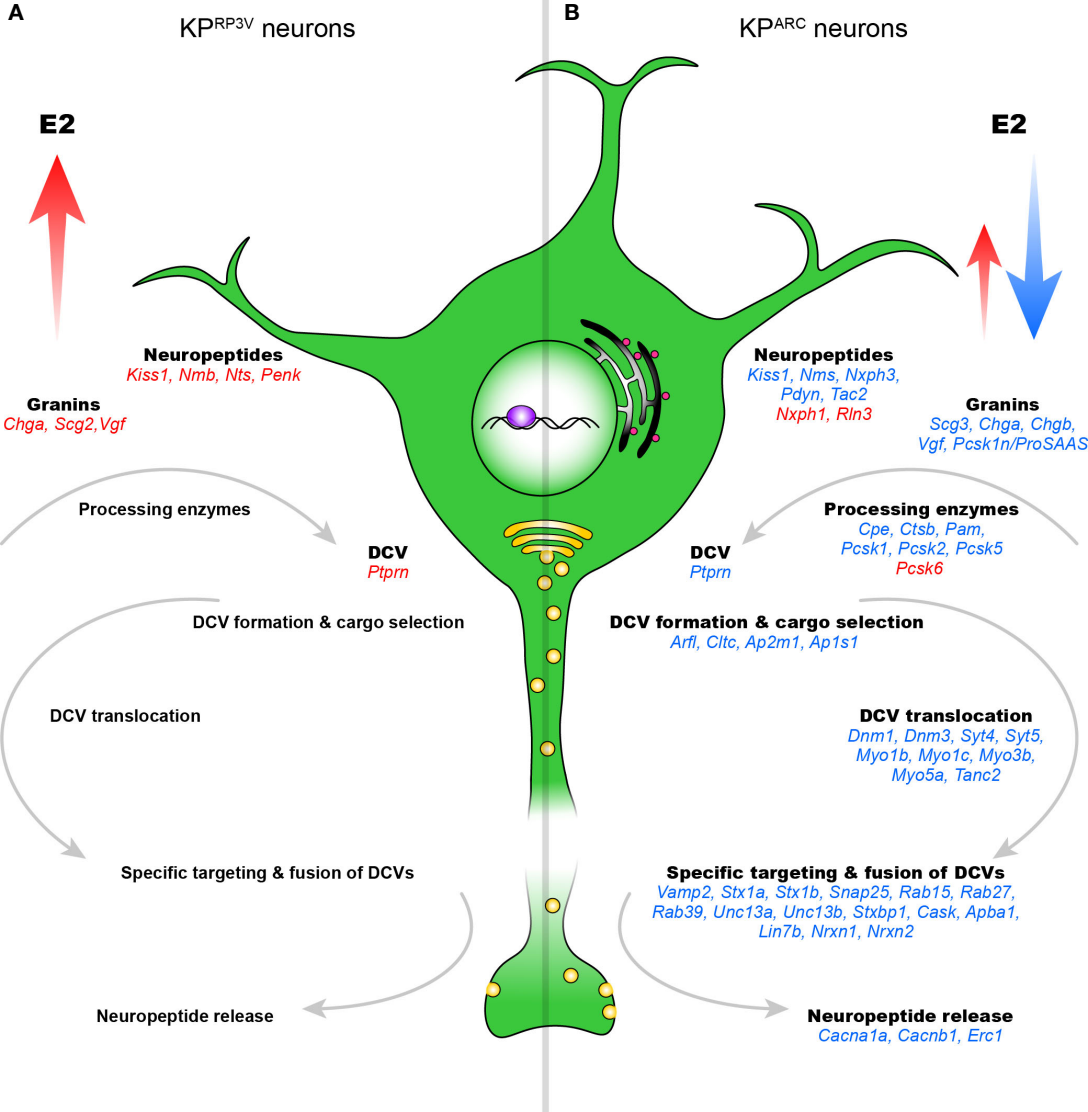
E2-dependent elements of the regulated secretory pathway in KP^RP3V^ and KP^ARC^ neurons. E2-regulated genes involved in neuropeptide secretion are shown in KP^RP3V^ **(A)** and KP^ARC^ neurons **(B)**. Genes in red and blue are up- and downregulated, respectively. DCV, dense-core vesicle.

In KP^RP3V^ neurons, we identified seven E2-regulated neuropeptide and granin genes. Transcriptional activation of *Nmb, Kiss1, Nts* and *Penk* was significant (**Figure 4A**), and these neuropeptide genes were ranked first, eleventh, seventeenth and fiftieth in the list of E2-regulated genes. E2-induced increase of *Pnoc, Prok2* and *Cartpt* did not reach statistical significance. KP^RP3V^ neurons highly expressed *Cartpt, Kiss1, Nmb, Nts* and *Penk*, while *Gal* was expressed moderately in OVX mice with E2 replacement. Neuropeptide precursor proteins are transported from the endoplasmic reticulum to the trans-Golgi network, where they are sorted and packed into DCVs. We showed upregulation of three granin genes, namely *Chga, Scg2*, *Vgf* and another gene of the secretory pathway, *Ptprn* (**Figure 4A**). Maturation of neuropeptides requires peptide bond cleavages in precursor molecules. E2 stimulated transcription of *Cpe, Pam* and *Pcsk2* prohormone processing enzymes, but the changes did not reach statistical significance (p.adj<0.05). Genes related to DCV translocation, transport and fusion were not regulated.

### E2 inhibits neuropeptide precursors, granins, processing enzymes and multiple secretory pathway genes in KP^ARC^ neurons

In KP^ARC^ neurons, we found transcriptional inhibition of five co-expressed neuropeptide precursor genes including *Kiss1, Nms, Nxph3, Pdyn* and *Tac2* (**Figure 4B**). We showed downregulation of five members of the granin family including *Scg3, Chga, Chgb, Vgf*, *Pcsk1n/ProSAAS* and another gene, *Ptprn* (**Figure 4B**). Neuropeptide maturation takes place in DCVs. E2 decreased mRNA expression of six processing enzymes including *Cpe, Ctsb, Pam, Pcsk1, Pcsk2* and *Pcsk5*, whereas the protease, *Pcsk6*, showed increased expression (**Figure 4B**). DCV formation and cargo selection depend on ADP-ribosylation factor 1 (*Arf1*) and components of the coat machinery. In KP^ARC^ neurons, E2 decreased transcription of *Arf1*, *Cltc, Ap2m1* and *Ap1s1*. From the trans-Golgi network, DCVs move towards the plasma membrane to release their content. Translocation relies upon dynamins, syntaxins, scaffolding and myosin motor proteins. In KP^ARC^ neurons, E2 downregulated mRNA expression of a large number of genes encoding dynamins (*Dnm1, Dnm3*), synaptotagmins (*Syt4, Syt5*) myosin (*Myo1b, Myo1c, Myo3b, Myo5a*) and scaffolding (*Tanc2*) proteins (**Figure 4B**). The SNARE complex and accessory factors are required for specific targeting and fusion of DCVs with the plasma membrane. E2 suppressed transcription of genes encoding components of the SNARE complex (*Vamp2*, *Stx1a, Stx1b*, *Snap25*) and accessory factors (*Rab15, Rab27, Rab39*, *Unc13a, Unc13b*). The DCV fusion machinery is linked to the Munc18-1/CASK/Mint1/Lin7b organizer complex, which binds to synaptic adhesion molecules neurexins. E2 downregulated constituents of the organizer complex [*Stxbp1* (coding Munc18-1), *Cask, Apba1* (coding Mint1), *Lin7b*] and neurexins (*Nrxn1, Nrxn2*) (**Figure 4B**). Cav2.1 and Cav2.2 channels orchestrate synchronous release of neuropeptides and neurotransmitters in most synapses. E2 downregulated *Cacna1a* (Cav2.1) and auxiliary Cav subunit *Cacnb1*, among others. Rab3-interacting proteins (RIMs), chief organizers of the active zone, are linked to Cav2.1 *via* RIM-binding protein encoded by *Erc1*, which was also suppressed by E2.

### Transcription factors show markedly different estrogenic regulation

**Figure 5:**
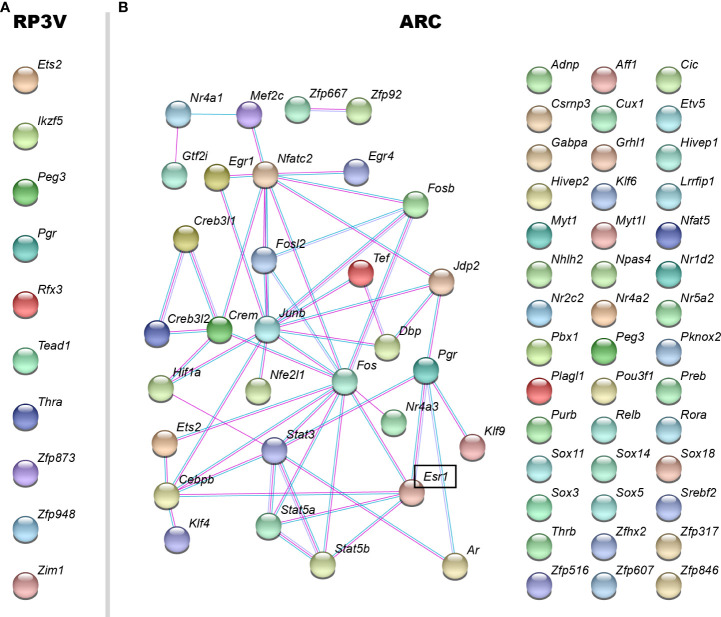
Protein interaction map of E2-dependent transcription factors in KP^RP3V^ and KP^ARC^ neurons. Based on the STRING database, protein interaction map of E2-regulated transcription factors was built using stringent settings (interaction source: experiment and databases, minimum required interaction score: medium confidence). STRING identified no potential interactions in KP^RP3V^ neurons **(A)**. In contrast, STRING found potential interactions between 31 E2-dependent transcription factors in KP^ARC^ neurons **(B)**. Genes in bold are regulated in both cell types. *Esr1*, which encodes ERα is in bold and framed.

Strikingly different transcriptional responses to E2 in KP^RP3V^ and KP^ARC^ neurons prompted us to determine the number of E2-dependent transcription factors. In KP^RP3V^ neurons, E2 regulated ten transcription factors. Based on the result of a recent publication (33), none of them interacted with ERα. In accord, STRING predicted no protein-protein interaction among them (**Figure 5A**). Neither transcriptional regulators, nor lncRNAs displayed E2-dependent expression in KP^RP3V^ neurons.

In KP^ARC^ neurons, E2 regulated mRNA expression of seventy transcription factors. E2-dependent transcription factors included nuclear hormone receptors (*Pgr, Rora, Thrb, Nr1d2, Nr2c2, Nr4a2, Nr5a2*, *Ar, Esr1, Nr4a1, Nr4a3*), homeobox proteins (*Adnp, Cux1, Pbx1, Pknox2, Zfhx2*), subunits of the AP-1 complex (*Fos, Junb, Jdp2*) and zinc finger proteins (*Hivep1, Zfp317*), among others. Of note, eight transcription factors including *Cebpb, Cic, Cux1, Fosl2, Gtf2i, Hivep1, Hivep2, Junb* were able to interact with ERα according to recently published data (33). To build a protein interaction map of E2-dependent transcription factors including putative interactions with ERα, we used the STRING database (34). Using strict settings (interaction source: experiment and databases, minimum required interaction score: medium confidence), STRING predicted thirty-four protein-protein interactions between thirty-one transcription factors (**Figure 5B**). According to STRING, five transcription factors can interact with ERα. The STRING protein interaction map predicted hubs in the network including ERα, Fos, Junb and Nfatc2.

In addition, E2 also modulated mRNA expression of genes encoding transcriptional regulators, chromatin modifiers and regulatory lncRNAs in KP^ARC^ neurons. E2 upregulated components of the SWI/SNF (*Smarca2, Smarca4, Smarcd3, Bicral*) and ATRX : DAXX (*Atrx, Daxx*) chromatin remodelling complexes, histone methyltransferases (*Kmt2e, Wdr82*) and deacetylases (*Hdac11*). E2 downregulated some transcriptional repressors (*Gatad2a, Trps1, Zfp219*), DNA methyltransferases (*Dnmt3a*) and histone deacetylases (*Hdac3, Hdac9*). Among them, one transcriptional coregulator (*Ncoa6*) and several repressors/activators (*Atrx, Gatad2a, Nrip1, Smarca2, Smarca4, Trim24, Trps1*) may interact with ERα. E2 also modified expression of numerous regulatory lncRNAs in KP^ARC^ neurons as described previously (17).

## Discussion

### E2 evokes different transcriptional responses in preoptic and arcuate kisspeptin neurons

To our knowledge, this is the first comprehensive study to examine and compare E2-driven transcriptional responses in KP^ARC^ and KP^RP3V^ neurons in the same animals. Here, we improved the power of DeSeq2 analysis by filtering out low-expression genes using a cutoff of basemean>20. This has resulted in a list of 222 and 1583 E2-regulated genes expressed at medium or high level in KP^RP3V^ and KP^ARC^ neurons, respectively. The highly different numbers of E2-dependent genes in the two kisspeptin neuron populations suggest that KP^ARC^ neurons are much more responsive to E2 treatment than KP^RP3V^ neurons in our model.

The goal of this study was to identify E2-regulated genes in KP^RP3V^ and KP^ARC^ neurons. In our recent study on the E2-dependent genes of KP^ARC^ neurons (17), we have justified the choice of non-physiological animal models (surgical OVX, followed by E2 substitution) to achieve this goal. Treatment of OVX mice with E2 or vehicle generates two well-defined experimental groups with little biological variations and large differences in the E2-dependent transcriptome profiles. In addition, using a single transcriptome snapshot the different regulatory dynamics of E2 dependent transcripts would also cause interpretation problems, complicated further by the cyclic presence of progesterone effects we could eliminate using our non-physiological models. Transcriptional responses in KP^ARC^ and KP^RP3V^ neurons to E2 in our model allowed us to identify E2-dependent genes and to compare their changes in the two kisspeptin neuronal populations. Of course, the gene expression profile of OVX+E2 mice exposed to high levels of E2 for 4 days is unlikely to mirror physiological conditions produced by peak E2 levels. We note that OVX rodents treated with constant E2 display a late afternoon LH surge which repeats daily at the same time (35). While high levels of E2 cause negative feedback on serum LH levels in our OVX+E2 animal model killed in the morning, transcriptomic changes which take place in response to E2 in KP^RP3V^ neurons (e.g. induction of *Kiss1*) may already be relevant to E2 positive feedback on LH secretion which is expected to occur in the late afternoon and requires a circadian signal as well. Future comparison of E2-treated animals in the morning with the late afternoon surging model will be particularly interesting in order to separate the activity-dependent regulatory changes from the E2-dependent ones in the transcriptome of KP^RP3V^ neurons.

We identified ninety-six overlapping E2-dependent genes including sixty-two genes with the same and thirty-four with opposite regulation. The sixty-two genes represent the common E2-dependent genes in hypothalamic kisspeptin neurons. Common upregulated genes encode three hormone receptors (*Ghsr, Pgr, Npr2*) indicating that ghrelin (and growth hormone), progesterone and natriuretic peptide may exert regulatory effects on estrogen feedback *via* acting on both kisspeptin cell populations. This notion is in accord with previously published data about the regulatory role of these hormones on kisspeptin neurons (36–38). Other common E2-dependent genes include *Gad2*, transcriptional activation of which may results in increased GABA synthesis in kisspeptin neurons. Of note, GABA exerts excitatory actions on GnRH neurons (39). The presence and estrogen-dependent regulation of *Gad2* in KP^ARC^ neurons is particularly interesting. Although the glutamatergic (Vglut2) phenotype of these neurons has been well-established (10, 40, 41), earlier *in situ* hybridization studies have already raised the possibility that a subpopulation may exhibit a mixed GABAergic/glutamatergic phenotype (10). RNAscope experiments recently revealed co-expression of vGAT and VGluT2 genes in the same cells (42), although functional studies indicate that KP^ARC^ neurons release glutamate but not GABA (43, 44). Opposite regulation by E2 characterizes a set of genes in KP^RP3V^ and KP^ARC^ neurons. This set contains *Kiss1*, *Vgf*, *Chrna7* and *Tmem35a*. So far, the role of Vgf and cholinergic transmission has not been described in estrogen feedback.

### Estrogens increase transcription of a set of neuropeptides in KP^RP3V^ neurons

Peptidergic transmission plays central role in the function of KP^RP3V^ neurons. We found upregulation of four co-expressed neuropeptides including *Kiss1, Nmb, Nts* and *Penk* (p.adj<0.05). Further, highly increased expression of three additional neuropeptides, namely *Pnoc, Prok2* and *Cartpt*, did not reach statistical significance (p.adj>0.05) in our study.

Of note, transcriptional activation of a set of neuropeptides occurs in response to E2 in KP^RP3V^ neurons. Among E2-dependent genes, *Nmb* showed the most robust response to E2 in our study. Neuromedin B stimulates GnRH release from hypothalamic extracts, and increases plasma LH level after intracerebroventricular administration (45). It has already been implicated in estrogen feedback as GnRH neurons express receptors for neuromedin B (46). Robust upregulation of *Nmb* supports the notion that neuromedin B may be an important regulator of positive estrogen feedback acting in concert with kisspeptins and other upregulated neuropeptides. We indicate that E2 also increases *Nts* expression in KP^RP3V^ neurons. According to a previous study, E2 induces *Nts* expression in the AVPV, and blockade of neurotensin signaling reduces the LH surge (47). Although GnRH neurons express *Ntsr2*, central administration of neurotensin does not induce LH surge. No co-expression of *Kiss1* and *Nts* has been detected by double-label ISH (47). We also detected expression of neurotensin receptors (*Ntsr1, Ntsr2*) in KP^RP3V^ neurons suggesting that neurotensin signaling plays a role in the communication between KP^RP3V^ neurons, in addition to signaling towards GnRH neurons. We also found that E2 enhanced *Penk* expression. In accord with this finding, a previous paper proves co-expression of kisspeptin and met-enkephalin in the AVPV (6). It is tempting to speculate that increased transcription of a set of neuropeptide genes including *Kiss1, Nmb, Nts* and *Penk* in KP^RP3V^ neurons might act in synergy to trigger the LH surge during positive feedback.

Following their synthesis, neuropeptide precursors undergo processing and transport prior to secretion. Granins, major constituents of DCV intravesicular matrix, bind Ca^2+^ and aggregate at acidic pH (48), which is considered to be the driving force of DCV biogenesis. We provide evidence that estrogens activate transcription of *Chga, Scg2*, and *Vgf*. Insulinoma-asssociated (Ia-2) protein is involved in the transcriptional control of DCV biogenesis (49). E2 activates *Ptprn* encoding Ia-2 protein, which may result in elevated DCV biogenesis.

A recent, elegant paper published the active translatome and its estrogenic regulation in AVPV kisspeptin neurons (50). The authors used the p<0.05 criterion and claimed 683 differentially expressed transcripts. Comparison of our results to the presented set of differentially expressed transcripts resulted in 52 overlapping genes in the two studies. Common E2-regulated genes included 13 genes with statistical significance (p.adj<0.05) in both studies (*Scg2, App, Maged1, Nap1l5, Itm2c, Calm1, Ptprn, Ckb, Zcchc12, Vgf, Gad2, Kiss1, Penk*) and 39 additional genes with statistical significance in our study and with marked difference without reported statistical significance in the study by Stephens and Kauffman (*C1ql2, Nmb, Sytl4, Crtac1, Tmem35a, Syt6, Fhod3, Aqp5, Maob, Brinp2, Pgr, Map3k15, Ghsr, Nxn, Nell2, Mgat1, Hcn1, Cadm1, Phyhipl, Fgd3, Pcp4, Rap1gap, Ipo5, Nts, Enah, Ptpn5, Usp48, Nrip1, Npr2, Tmcc3, Hs3st5, Hpcal1, Ngb, Flrt3, Thsd7b, Pgr15l, Peg10, Npy2r, Slc17a8).* The four upregulated neuropeptides (*Kiss1, Nmb, Nts* and *Penk*) in our study mentioned previously were among the overlapping genes. Although our study and the paper by Stephens and Kauffman (50) used dissimilar methodologies (Kiss1-Cre/ZsGreen vs. *Kiss1*Cre/Ribotag mice, 4-day E2 treatment vs. two sequential E2 treatments with different hormone regimen) and targeted distinct RNA populations (total RNA vs. ribosome bound mRNA) for sequencing, more than fifty genes with almost identical estrogenic regulation were observed in preoptic kisspeptin neurons in the two studies.

### Estrogens inhibit transcription of neuropeptide precursor, granin, processing enzyme and DCV related genes in KP^ARC^ neurons

We find that both kisspeptin neuron populations possess unique neuropeptide profiles that are highly regulated by E2. In KP^ARC^ neurons, besides *Kiss1* we show four more co-expressed neuropeptide genes including *Nms, Nxph3, Pdyn, Tac2* that are all suppressed. We demonstrate that estrogens suppress not only genes encoding neuropeptide precursors, but granins, processing enzymes and multiple elements of the regulated secretory pathway. Along with granins and their processing enzymes, neuropeptide precursors are transported from the endoplasmic reticulum to the trans-Golgi network, where they are sorted and packed into DCVs. E2 inhibits several highly expressed granin genes including *Chga, Chgb, Pcsk1n, Scg3*, *Vgf* and Ia-2 protein coding gene *Ptprn* that may lead to decreased DCV biogenesis. E2 inhibits six protease genes including *Cpe, Ctsb, Pam, Pcsk1, Pcsk2, Pcsk5* that can be involved in the maturation of co-expressed neuropeptides in KP^ARC^ neurons. DCVs move towards the plasma membrane to release their content. This translocation relies upon dynamins, syntaxins, scaffolding and myosin motor proteins. E2 inhibits transcription of several genes encoding dynamins, synaptotagmins, myosins and scaffolding proteins. Specific targeting and fusion of DCVs with the plasma membrane requires concerted action of SNARE proteins and accessory factors. We show that E2 inhibits transcription of genes encoding multiple components of the SNARE complex and accessory proteins. The fusion machinery is linked to the organizer complex, which binds to synaptic adhesion molecules neurexins. E2 suppresses constituents of the Munc18-1/CASK/Mint1/Lin7b organizer complex and neurexins. Cav2.1 and Cav2.2 channels, which orchestrate synchronous release of neuropeptides and neurotransmitters were also downregulated. With the above findings, we provide evidence that an intricate E2-driven transcriptional regulatory mechanism exists in KP^ARC^ neurons, which can provide coordinated suppression of multiple elements of the secretory pathway. Of note, we can’t exclude the possibility that a minor portion of fluorescently labelled cells does not express *Kiss1* at the time of sample collection. We also do not know that which set of E2-dependent genes is expressed in a given cell due to cell samples containing 300 pooled kisspeptin neurons.

### Mechanisms underlying the transcriptional changes

In line with previous papers we found that the effects of estrogens are mediated solely by ERα in both arcuate and preoptic kisspeptin neurons (51). We detected abundant expression of *Esr1*, which encodes ERα. Neither *Esr2* nor *Gper1* encoding ERβ and G-protein coupled estrogen receptor, respectively, showed expression in KP^RP3V^ and KP^ARC^ neurons. Our results confirm that E2-driven transcriptional effects in kisspeptin neurons are mediated solely by ERα. Multiple ERα-driven mechanisms (52, 53) that operate in hypothalamic kisspeptin neurons were inseparable in our study. ERα can bind in *cis* (chromatin association *via* direct DNA binding at ERE) or in *trans* (chromatin association *via* binding to other transcription factors) at enhancers. Enhancer activation requires cooperative recruitment of multiple transcription factors and their cofactors. Approximately 200-300 transcription factors are expressed in each cell type (54). Expression of ten transcription factors was E2-dependent in KP^RP3V^ neurons. In contrast, expression of seventy was E2-dependent in KP^ARC^ neurons, eight of which interacted with ERα. The STRING database predicted an intricate network of transcription factors in KP^ARC^ neurons. In addition, E2 regulated several transcriptional regulators, chromatin modifiers and regulatory lncRNAs (17) adding another layer of complexity to the ERα mediated transcriptional mechanism in KP^ARC^ neurons. The complex, multi-layered transcriptional regulatory mechanism allows KP^ARC^ neurons to respond and integrate humoral and neuronal inputs that influence reproduction, potentially including metabolic, circadian and stress-related cues. The less complex estrogen-dependent mechanisms revealed in KP^RP3V^ neurons suggest a less integrative role of this population at least in our model.

## Data availability statement

The data for KPRP3V neuron sequencing presented in the study are deposited in the BioProject repository, accession number of PRJNA847063. The data for KPARC neuron sequencing presented in the study have already been released: BioProject repository, accession number PRJNA686688.

## Ethics statement

The animal study was reviewed and approved by Institutional Animal Care and Use Committee.

## Author contributions

Conceptualization, BG, ST, KS, ER, NS, SP, WHC, EH, and MS; Methodology, BG, ST, KS, ER, NS, SP, EH, and MS; Investigation, BG, ST, KS, ER, NS, SP, WHC, EH, and MS; Writing-editing, BG, ST, KS, ER, NS, SP, WHC, EH, and MS; Funding acquisition and Supervision, KS and EH. All authors contributed to the article and approved the submitted version.

## Funding

The research leading to these results has received funding from the National Science Foundation of Hungary (K128317 and 138137 to EH and PD134837 to KS) and the Hungarian Brain Research Program (2017-1.2.1-NKP-2017-00002 to EH).

## Conflict of interest

The authors declare that the research was conducted in the absence of any commercial or financial relationships that could be construed as a potential conflict of interest.

## Publisher’s note

All claims expressed in this article are solely those of the authors and do not necessarily represent those of their affiliated organizations, or those of the publisher, the editors and the reviewers. Any product that may be evaluated in this article, or claim that may be made by its manufacturer, is not guaranteed or endorsed by the publisher.

## References

[1] de Roux N Genin E Carel JC Matsuda F Chaussain JL Milgrom E. Hypogonadotropic hypogonadism due to loss of function of the KiSS1-derived peptide receptor GPR54. Proc Natl Acad Sci USA. (2003) 100(19):10972–6. doi: 10.1073/pnas.1834399100 PMC196911 12944565

[2] Seminara SB Messager S Chatzidaki EE Thresher RR Acierno JS Jr Shagoury JK. The GPR54 gene as a regulator of puberty. N Engl J Med (2003) 349(17):1614–27. doi: 10.1056/NEJMoa035322 14573733

[3] Topaloglu AK Tello JA Kotan LD Ozbek MN Yilmaz MB Erdogan S. Inactivating KISS1 mutation and hypogonadotropic hypogonadism. N Engl J Med (2012) 366(7):629–35. doi: 10.1056/NEJMoa1111184 22335740

[4] Funes S Hedrick JA Vassileva G Markowitz L Abbondanzo S Golovko A. The KiSS-1 receptor GPR54 is essential for the development of the murine reproductive system. Biochem Biophys Res Commun (2003) 312(4):1357–63. doi: 10.1016/j.bbrc.2003.11.066 14652023

[5] d'Anglemont de Tassigny X Fagg LA Dixon JP Day K Leitch HG Hendrick AG. Hypogonadotropic hypogonadism in mice lacking a functional Kiss1 gene. Proc Natl Acad Sci USA. (2007) 104(25):10714–9. doi: 10.1073/pnas.0704114104 PMC1965578 17563351

[6] Porteous R Petersen SL Yeo SH Bhattarai JP Ciofi P de Tassigny XD. Kisspeptin neurons co-express met-enkephalin and galanin in the rostral periventricular region of the female mouse hypothalamus. J Comp Neurol (2011) 519(17):3456–69. doi: 10.1002/cne.22716 21800299

[7] Kallo I Vida B Deli L Molnar CS Hrabovszky E Caraty A. Co-Localisation of kisspeptin with galanin or neurokinin b in afferents to mouse GnRH neurones. J Neuroendocrinol (2012) 24(3):464–76. doi: 10.1111/j.1365-2826.2011.02262.x 22129075

[8] Clarkson J Herbison AE. Dual phenotype kisspeptin-dopamine neurones of the rostral periventricular area of the third ventricle project to gonadotrophin-releasing hormone neurones. J Neuroendocrinol (2011) 23(4):293–301. doi: 10.1111/j.1365-2826.2011.02107.x 21219482

[9] Kauffman AS Gottsch ML Roa J Byquist AC Crown A Clifton DK. Sexual differentiation of Kiss1 gene expression in the brain of the rat. Endocrinology (2007) 148(4):1774–83. doi: 10.1210/en.2006-1540 17204549

[10] Cravo RM Margatho LO Osborne-Lawrence S Donato J Jr. Atkin S Bookout AL. Characterization of Kiss1 neurons using transgenic mouse models. Neuroscience (2011) 173:37–56. doi: 10.1016/j.neuroscience.2010.11.022 21093546 PMC3026459

[11] Herbison AE. The gonadotropin-releasing hormone pulse generator. Endocrinology (2018) 159(11):3723–36. doi: 10.1210/en.2018-00653 30272161

[12] Sobrino V Avendano MS Perdices-Lopez C Jimenez-Puyer M Tena-Sempere M. Kisspeptins and the neuroendocrine control of reproduction: Recent progress and new frontiers in kisspeptin research. Front Neuroendocrinol (2022) 65:100977. doi: 10.1016/j.yfrne.2021.100977 34999056

[13] Campbell JN Macosko EZ Fenselau H Pers TH Lyubetskaya A Tenen D. A molecular census of arcuate hypothalamus and median eminence cell types. Nat Neurosci (2017) 20(3):484–96. doi: 10.1038/nn.4495 PMC5323293 28166221

[14] Moffitt JR Bambah-Mukku D Eichhorn SW Vaughn E Shekhar K Perez JD. Molecular, spatial, and functional single-cell profiling of the hypothalamic preoptic region. Science (2018) 362(6416). doi: 10.1126/science.aau5324 PMC6482113 30385464

[15] Garcia-Galiano D Pinilla L Tena-Sempere M. Sex steroids and the control of the Kiss1 system: developmental roles and major regulatory actions. J Neuroendocrinol (2012) 24(1):22–33. doi: 10.1111/j.1365-2826.2011.02230.x 21951227

[16] Gottsch ML Navarro VM Zhao Z Glidewell-Kenney C Weiss J Jameson JL. Regulation of Kiss1 and dynorphin gene expression in the murine brain by classical and nonclassical estrogen receptor pathways. J Neurosci (2009) 29(29):9390–5. doi: 10.1523/JNEUROSCI.0763-09.2009 PMC2819182 19625529

[17] Gocz B Rumpler E Sarvari M Skrapits K Takacs S Farkas I. Transcriptome profiling of kisspeptin neurons from the mouse arcuate nucleus reveals new mechanisms in estrogenic control of fertility. Proc Natl Acad Sci U.S.A. (2022) 119(27):e2113749119. doi: 10.1073/pnas.2113749119 35763574 PMC9271166

[18] Yeo SH Kyle V Morris PG Jackman S Sinnett-Smith LC Schacker M. Visualisation of Kiss1 neurone distribution using a Kiss1-CRE transgenic mouse. J Neuroendocrinol (2016) 28(11). doi: 10.1111/jne.12435 PMC5091624 27663274

[19] Molnar CS Kallo I Liposits Z Hrabovszky E. Estradiol down-regulates RF-amide-related peptide (RFRP) expression in the mouse hypothalamus. Endocrinology (2011) 152(4):1684–90. doi: 10.1210/en.2010-1418 21325049

[20] Schuierer S Carbone W Knehr J Petitjean V Fernandez A Sultan M. A comprehensive assessment of RNA-seq protocols for degraded and low-quantity samples. BMC Genomics (2017) 18(1):442. doi: 10.1186/s12864-017-3827-y 28583074 PMC5460543

[21] Dobin A Davis CA Schlesinger F Drenkow J Zaleski C Jha S. STAR: ultrafast universal RNA-seq aligner. Bioinformatics (2013) 29(1):15–21. doi: 10.1093/bioinformatics/bts635 23104886 PMC3530905

[22] Liao Y Smyth GK Shi W. featureCounts: an efficient general purpose program for assigning sequence reads to genomic features. Bioinformatics (2014) 30(7):923–30. doi: 10.1093/bioinformatics/btt656 24227677

[23] McCarthy DJ Chen Y Smyth GK. Differential expression analysis of multifactor RNA-seq experiments with respect to biological variation. Nucleic Acids Res (2012) 40(10):4288–97. doi: 10.1093/nar/gks042 PMC3378882 22287627

[24] Love MI Huber W Anders S. Moderated estimation of fold change and dispersion for RNA-seq data with DESeq2. Genome Biol (2014) 15(12):550. doi: 10.1186/s13059-014-0550-8 25516281 PMC4302049

[25] Benjamini Y Drai D Elmer G Kafkafi N Golani I. Controlling the false discovery rate in behavior genetics research. Behav Brain Res (2001) 125(1-2):279–84. doi: 10.1016/S0166-4328(01)00297-2 11682119

[26] Kanehisa M Goto S. KEGG: kyoto encyclopedia of genes and genomes. Nucleic Acids Res (2000) 28(1):27–30. doi: 10.1093/nar/28.1.27 10592173 PMC102409

[27] Tavazoie S Hughes JD Campbell MJ Cho RJ Church GM. Systematic determination of genetic network architecture. Nat Genet (1999) 22(3):281–5. doi: 10.1038/10343 10391217

[28] Wu T Hu E Xu S Chen M Guo P Dai Z. clusterProfiler 4.0: A universal enrichment tool for interpreting omics data. Innovation (NY) (2021) 2(3):100141. doi: 10.1016/j.xinn.2021.100141 PMC8454663 34557778

[29] Hu H Miao YR Jia LH Yu QY Zhang Q Guo AY. AnimalTFDB 3.0: a comprehensive resource for annotation and prediction of animal transcription factors. Nucleic Acids Res (2019) 47(D1):D33–8. doi: 10.1093/nar/gky822 PMC6323978 30204897

[30] Nilsson ME Vandenput L Tivesten A Norlen AK Lagerquist MK Windahl SH. Measurement of a comprehensive sex steroid profile in rodent serum by high-sensitive gas chromatography-tandem mass spectrometry. Endocrinology (2015) 156(7):2492–502. doi: 10.1210/en.2014-1890 25856427

[31] Bourgon R Gentleman R Huber W. Independent filtering increases detection power for high-throughput experiments. Proc Natl Acad Sci U.S.A. (2010) 107(21):9546–51. doi: 10.1073/pnas.0914005107 PMC2906865 20460310

[32] Sha Y Phan JH Wang MD. (2015). Effect of low-expression gene filtering on detection of differentially expressed genes in RNA-seq data, in: Annu Int Conf IEEE Eng Med Biol Soc, , Vol. 2015. pp. 6461–4. doi: 10.1109/EMBC.2015.7319872 PMC4983442 26737772

[33] Bi M Zhang Z Jiang YZ Xue P Wang H Lai Z. Enhancer reprogramming driven by high-order assemblies of transcription factors promotes phenotypic plasticity and breast cancer endocrine resistance. Nat Cell Biol (2020) 22(6):701–15. doi: 10.1038/s41556-020-0514-z PMC7737911 32424275

[34] Szklarczyk D Gable AL Nastou KC Lyon D Kirsch R Pyysalo S. The STRING database in 2021: customizable protein-protein networks, and functional characterization of user-uploaded gene/measurement sets. Nucleic Acids Res (2021) 49(D1):D605–12. doi: 10.1093/nar/gkaa1074 PMC7779004 33237311

[35] de la Iglesia HO Schwartz WJ. Minireview: timely ovulation: circadian regulation of the female hypothalamo-pituitary-gonadal axis. Endocrinology (2006) 147(3):1148–53. doi: 10.1210/en.2005-1311 16373412

[36] de Paula DG Bohlen TM Zampieri TT Mansano NS Vieira HR Gusmao DO. Distinct effects of growth hormone deficiency and disruption of hypothalamic kisspeptin system on reproduction of male mice. Life Sci (2021) 285:119970. doi: 10.1016/j.lfs.2021.119970 34562435

[37] Mohr MA Esparza LA Steffen P Micevych PE Kauffman AS. Progesterone receptors in AVPV kisspeptin neurons are sufficient for positive feedback induction of the LH surge. Endocrinology (2021) 162(11). doi: 10.1210/endocr/bqab161 PMC8423423 34379733

[38] Samson WK Alexander BD Skala KD Huang FL Fulton RJ. Central peptidergic mechanisms controlling reproductive hormone secretion: novel methodology reveals a role for the natriuretic peptides. Can J Physiol Pharmacol (1992) 70(5):773–8. doi: 10.1139/y92-102 1423020

[39] Moenter SM DeFazio RA. Endogenous gamma-aminobutyric acid can excite gonadotropin-releasing hormone neurons. Endocrinology (2005) 146(12):5374–9. doi: 10.1210/en.2005-0788 16123153

[40] Takacs S Bardoczi Z Skrapits K Gocz B Vaczi V Magloczky Z. Post mortem single-cell labeling with DiI and immunoelectron microscopy unveil the fine structure of kisspeptin neurons in humans. Brain Struct Funct (2018) 223(5):2143–56. doi: 10.1007/s00429-018-1610-8 29380121

[41] Ciofi P Leroy D Tramu G. Sexual dimorphism in the organization of the rat hypothalamic infundibular area. Neuroscience (2006) 141(4):1731–45. doi: 10.1016/j.neuroscience.2006.05.041 16809008

[42] Moore AM Lohr DB Coolen LM Lehman MN. Prenatal androgen exposure alters KNDy neurons and their afferent network in a model of polycystic ovarian syndrome. Endocrinology (2021) 162(11). doi: 10.1210/endocr/bqab158 PMC8402932 34346492

[43] Qiu J Nestor CC Zhang C Padilla SL Palmiter RD Kelly MJ. High-frequency stimulation-induced peptide release synchronizes arcuate kisspeptin neurons and excites GnRH neurons. Elife (2016) 5. doi: 10.7554/eLife.16246 PMC4995096 27549338

[44] Qiu J Rivera HM Bosch MA Padilla SL Stincic TL Palmiter RD. Estrogenic-dependent glutamatergic neurotransmission from kisspeptin neurons governs feeding circuits in females. Elife (2018) 7. doi: 10.7554/eLife.35656 PMC6103748 30079889

[45] Boughton CK Patel SA Thompson EL Patterson M Curtis AE Amin A. Neuromedin b stimulates the hypothalamic-pituitary-gonadal axis in male rats. Regul Pept (2013) 187:6–11. doi: 10.1016/j.regpep.2013.10.002 24120470

[46] Todman MG Han SK Herbison AE. Profiling neurotransmitter receptor expression in mouse gonadotropin-releasing hormone neurons using green fluorescent protein-promoter transgenics and microarrays. Neuroscience (2005) 132(3):703–12. doi: 10.1016/j.neuroscience.2005.01.035 15837132

[47] Dungan Lemko HM Naderi R Adjan V Jennes LH Navarro VM Clifton DK. Interactions between neurotensin and GnRH neurons in the positive feedback control of GnRH/LH secretion in the mouse. Am J Physiol Endocrinol Metab (2010) 298(1):E80–8. doi: 10.1152/ajpendo.00380.2009 PMC2806107 19861584

[48] Kim T Gondre-Lewis MC Arnaoutova I Loh YP. Dense-core secretory granule biogenesis. Physiol (Bethesda) (2006) 21:124–33. doi: 10.1152/physiol.00043.2005 16565478

[49] Harashima S Clark A Christie MR Notkins AL. The dense core transmembrane vesicle protein IA-2 is a regulator of vesicle number and insulin secretion. Proc Natl Acad Sci USA. (2005) 102(24):8704–9. doi: 10.1073/pnas.0408887102 PMC1150807 15939893

[50] Stephens SBZ Kauffman AS. Estrogen regulation of the molecular phenotype and active translatome of AVPV kisspeptin neurons. Endocrinology (2021) 162(9). doi: 10.1210/endocr/bqab080 PMC8286094 33856454

[51] Smith JT Cunningham MJ Rissman EF Clifton DK Steiner RA. Regulation of Kiss1 gene expression in the brain of the female mouse. Endocrinology (2005) 146(9):3686–92. doi: 10.1210/en.2005-0488 15919741

[52] Gronemeyer H Gustafsson JA Laudet V. Principles for modulation of the nuclear receptor superfamily. Nat Rev Drug Discovery (2004) 3(11):950–64. doi: 10.1038/nrd1551 15520817

[53] Farcas AM Nagarajan S Cosulich S Carroll JS. Genome-wide estrogen receptor activity in breast cancer. Endocrinology (2021) 162(2). doi: 10.1210/endocr/bqaa224 PMC7787425 33284960

[54] Vaquerizas JM Kummerfeld SK Teichmann SA Luscombe NM. A census of human transcription factors: function, expression and evolution. Nat Rev Genet (2009) 10(4):252–63. doi: 10.1038/nrg2538 19274049

